# Level of hM4D(Gi) DREADD Expression Determines Inhibitory and Neurotoxic Effects in the Hippocampus

**DOI:** 10.1523/ENEURO.0105-21.2021

**Published:** 2021-11-02

**Authors:** Marie-Gabrielle Goossens, Lars Emil Larsen, Marijke Vergaelen, Wytse Wadman, Chris Van den Haute, Wayra Brackx, Silke Proesmans, Jana Desloovere, Emma Christiaen, Erine Craey, Christian Vanhove, Kristl Vonck, Paul Boon, Robrecht Raedt

**Affiliations:** 1 4BRAIN, Department Head and Skin, Ghent University, Ghent 9000, Belgium; 2 Laboratory for Neurobiology and Gene Therapy, Center for Molecular Medicine and Leuven Brain Institute, Katholieke Universiteit Leuven, Leuven 3000, Belgium; 3 Leuven Viral Vector Core, Center for Molecular Medicine, Katholieke Universiteit Leuven, Leuven 3000, Belgium; 4 Department of Basic and Applied Medical Sciences, Ghent University, Ghent 9000, Belgium; 5 Medisip, Department Electronics and Information Systems, Ghent University, Ghent 9000, Belgium

**Keywords:** chemogenetics, DREADD, hippocampus, neurotoxicity, rat, viral vector

## Abstract

Selective neuromodulation using designer receptors exclusively activated by designer drugs (DREADDs) has become an increasingly important research tool, as well as an emerging therapeutic approach. However, the safety profile of DREADD expression is unknown. Here, different titers of adeno-associated viral (AAV) vector were administered in an attempt to vary total expression levels of the inhibitory DREADD hM4D(Gi) in excitatory hippocampal neurons. Male Sprague Dawley rats were injected with AAV2/7 encoding DREADD-mCherry, DREADD, or mCherry. Pronounced neuronal loss and neuroinflammatory reactions were observed after transduction with the high titer DREADD AAV, which also resulted in the highest DREADD expression levels. No such effects were observed in the mCherry control group, despite an equally high titer, nor in conditions where lower viral vector titers were injected. In the high titer DREADD conditions, dentate gyrus (DG) evoked potentials were inhibited on clozapine-induced activation of hM4D(Gi), while in low titer conditions DG evoked potentials were enhanced. Recordings of single neuronal activity nevertheless indicated a reduction in spontaneous firing of granule cell layer neurons. Our results indicate that prolonged, high levels of DREADD expression can have neurotoxic effects and that chemogenetic suppression of excitatory hippocampal neurons can paradoxically enhance DG evoked potentials.

## Significance Statement

Designer receptors exclusively activated by designer drugs (DREADDs) are engineered receptors that can be used to selectively modulate specific groups of cells. Especially in neuroscience, DREADDs are widely adopted. However, there is not much known on their safety profile. Here, we assess the effect of different expression levels of the DREADD hM4D(Gi) by varying the titer of the adeno-associated viral (AAV) vector used to transduce specific neurons in the rat’s brain. We found that high expression levels result in strong neuromodulatory effects, but also induce neuronal loss and tissue damage. Less pronounced, non-toxic expression levels paradoxically seem to display opposite neuromodulatory effects at network level.

## Introduction

Designer receptors exclusively activated by designer drugs (DREADDs) are engineered human muscarinic receptors that are no longer responsive to their original ligand, but can instead be selectively activated by exogenous actuators such as clozapine-*N*-oxide, deschloroclozapine, JHU37152, JHU37160 and low doses of clozapine (Armbruster et al., 2007; Gomez et al., 2017; Bonaventura et al., 2019; Nagai et al., 2020). DREADD variants include hM3D(Gq), an excitatory receptor that acts via the G_αq_ pathway and is used to enhance neuronal firing, and hM4D(Gi), an inhibitory receptor that activates the G_αi_ pathway, resulting in decreased neuronal firing and synaptic silencing (Armbruster et al., 2007; Alexander et al., 2009). Especially in neuroscience, DREADDs are widely adopted (Roth, 2016). Selective modulation of neuronal excitability by chemogenetics has become an increasingly important research tool, as well as an emerging therapeutic approach (Sternson and Roth, 2014; English and Roth, 2015). DREADDs can be used to selectively activate or inhibit a specific type of neuron to elucidate its function and modulate its activity. Currently, DREADD research ranges from spinal cord injury to addiction, neurodegenerative diseases and focal epilepsy (Roth, 2016). If a pathologic condition is associated with an increase or decrease in activity of specific cells, chemogenetic modulation of these cell types could help to restore a normal condition. It was recently demonstrated that selective hM4D(Gi) expression in principal hippocampal neurons results in seizure suppressing effects, both in chronic mouse and rat models for temporal lobe epilepsy (Wang et al., 2018; Desloovere et al., 2019; Goossens et al., 2021).

In the view of future clinical translation, DREADD gene delivery is obtained through a viral vector carrier, most commonly a recombinant adeno-associated viral (AAV) vector (Urban and Roth, 2015). Selective expression can be obtained using an adequate AAV serotype and a cell type-specific promoter, such as the CaMKIIα promoter to selectively target excitatory neurons (Haery et al., 2019). In contrast to AAV vectors, which are generally considered safe and are currently being tested in several Phase I and II clinical trials, not much is known on the safety profile and recommended viral doses of the DREADD technology itself (Wang et al., 2019). The viral titers and injected volumes reported in literature on preclinical DREADD research in rodents are highly variable, ranging from E + 9 to E + 13 vector genomes (vg)/ml and 0.01–5 μl per injection site, respectively (Wirtshafter and Stratford, 2016; Rorabaugh et al., 2017; Wunsch et al., 2017; Maharjan et al., 2018; Panoz-Brown et al., 2018; Dygalo et al., 2020; Stevens et al., 2020). The minimal DREADD expression level (determined by the AAV serotype and titer, the promoter sequence and the injected volume) required to result in effective neuromodulation is unknown. On the other hand, it is not known whether there is a critical limit for DREADD expression that once surpassed, leads to, e.g., neurotoxicity. Although neurotoxicity with chronic hM4D(Gi) expression has been reported during a study in a rat model for epilepsy (Goossens et al., 2021), no explicit safety issues have been reported with AAV-mediated DREADD expression in other animal studies. Nevertheless, the absence of reports of toxicity does not imply the absence of toxicity.

Here, we focused on AAV-mediated hM4D(Gi) expression when specifically targeting excitatory (CaMKIIα-expressing) hippocampal neurons, i.e., the granule cells in the dentate gyrus (DG) and the pyramidal cells in the CA regions. This approach represents one of the chemogenetic applications with therapeutic potential, e.g., in epilepsy (Desloovere et al., 2019; Chen et al., 2020; Goossens et al., 2021). The aim of this study was to vary hM4D(Gi) expression levels in hippocampal neurons by injecting different viral vector titers. Possible neurotoxic effects were assessed by means of anatomic T2 MR images and histologic analysis. The electrophysiological effect of DREADD activation was evaluated by means of DG evoked field potentials (EPs), and in a subset of animals, by measuring the spontaneous activity of neurons in the DG.

## Materials and Methods

### Animals

Thirty-six adult male Sprague Dawley rats (Envigo) were used. Animals were treated according to European guidelines (directive 2010/63/EU) and the protocol was approved by the local Ethics Committee on Animal Experiments of Ghent University (ECD 16/31). Animals were kept under environmentally controlled conditions (20–24°C and 20–60% relative humidity) with a fixed 12/12 h light/dark cycle [zeitgeber time (ZT)0 = 9 A.M.]. The animals were group-housed in Type IV cages (Tecniplast) on wood-based bedding (Carfil). Cages were enriched with gnawing woods (M-brick; Carfil), paper nesting material (Nesting; Carfil), and a cardboard tunnel (cardboard tunnel 5 mm; Carfil). Water and food (Rats and Mice Maintenance; Carfil) were provided *ad libitum*. Before the start of the experimental procedures, animals were handled 10 min/d for 5 d in total to accustom them to human contact.

### Recombinant AAV production and purification

Viral vectors were kindly provided by the Katholieke Universiteit Leuven vector core unit (www.lvvc.be). Vector production and purification was performed as previously described (Van Der Perren et al., 2011). Plasmids include the constructs for the AAV2/7 serotype, the transfer plasmid for the fluorescent tag mCherry, the inhibitory DREADD hM4D(Gi) or the fusion protein hM4D(Gi)-mCherry transgene under control of the mouse CamKIIα 0.4 promoter as well as the pAdvDeltaF6 adenoviral helper plasmid. Real-time polymerase chain reaction analysis was used for vector genome copy determination.

### Intrahippocampal AAV vector injection

Animals (298 ± 25 g body weight) were injected with AAV vector. An overview of the groups, injected vectors and titers is given in Table 1. For ease of reading, group names are denoted as the viral titer rounded to the nearest power of ten. Vectors were stored at −80°C before use and diluted using sterile PBS. Animals were allocated to groups at random using a random number generator.

**Table 1:** Overview of injected AAV vectors, corresponding titer, and number of animals per group used in this study

Group	AAV vector	Titer (vg/ml)	Total viral load (vg)	Number of animals
D13	AAV 2/7- CamKIIα(0.4)-intron-hM4D(Gi)	8.1E + 12	4.9E + 10	8
M13	AAV 2/7- CamKIIα(0.4)-intron-mCherry	2.1E + 13	1.2E + 11	3
DM13	AAV 2/7- CamKIIα(0.4)-intron-hM4D(Gi)-mCherry	2.8E + 13	1.7E + 11	10
DM12	AAV 2/7- CamKIIα(0.4)-intron-hM4D(Gi)-mCherry	9.3E + 11	5.6E + 09	9
DM11	AAV 2/7- CamKIIα(0.4)-intron-hM4D(Gi)-mCherry	2.8E + 11	1.7E + 09	6

D = DREADD, M = mCherry, DM = DREADD-mCherry, vg = vector genomes.

Animals were anesthetized with a mixture of isoflurane (Isoflo, 5% for induction, 2% for maintenance; Zoetis) and medical oxygen. After exposure of the skull, two small burr holes were made. Using a 32-gauge needle, a 5-μl Hamilton syringe (Neuros syringe, 65460-03; Hamilton Company), and an automated injection system (Quintessential Stereotaxic Injector; Stoelting), AAV was injected at two sites in the right hippocampus using these respective stereotactic coordinates: anteroposterior (AP): −3.8 and −5.2 mm to bregma, mediolateral (ML): −2.2 and −5.0 mm to bregma, dorsoventral (DV): −3.2 and −7.0 mm to dura. Per injection site, 3 μl of AAV was injected at a flow rate of 0.3 μl/min. After each injection, the syringe was left in place for an additional 5 min. Afterwards, the skin was closed, rats were subcutaneously injected with the nonsteroidal anti-inflammatory drug meloxicam (NSAID; 1 mg/kg metacam; Boehringer Ingelheim) and lidocaine (2% xylocaine gel; AstraZeneca) was locally applied to the wound to minimize discomfort. In between injections, syringes were cleaned according to the guidelines of the manufacturer.

### Anatomical MRI scans

Animals from the D13 group were scanned 3, 5, and 12 weeks after AAV injection. A subset (*n*_D13_ = 4) of the animals was also scanned at 21 weeks. The other animals (*n*_DM13_ = 10, *n*_DM12_ = 6, *n*_DM11_ = 6, *n*_M13_ = 3) were scanned five weeks after injection. Anatomical MR images were acquired using a lab animal sized Bruker BioSpin PharmaScan 7T system (Bruker) with a transmit volume coil (Rapid Biomedical) and an actively-decoupled rat head surface coil (Rapid Biomedical). TurboRARE T2-weighted images (TR 3661 ms, TE 37 ms, 30 slices, FOV 35 × 35 mm^2^, in-plane slice resolution 109 × 109 μm^2^, slice thickness 0.6 mm, acquisition time 9 min 46 s) were acquired. Animals were anesthetized as described earlier and equipped with a heating pad and pressure sensor to control body temperature and respiration during scanning.

### DG EP recording *in vivo*

EPs were recorded four to five weeks after AAV injection. Animals were anaesthetized as described earlier and a unilateral recording and stimulation electrode were implanted in the right hippocampus and the right perforant path, respectively. Body temperature was kept constant using a heating pad. After exposure of the skull, small burr holes were made: two for the epidural ground/reference electrodes above right and left frontal cortex, respectively, one for the recording and one for the stimulation electrode. Epidural electrodes were custom-made by attaching an insulated copper wire to an anchor screw (1.75-mm diameter; Invivo1). Depth recording and stimulation electrodes were custom-made by twisting two polyimide-coated stainless-steel wires [70-μm bare diameter (California Fine Wire) and 125-μm bare diameter (Science Products), respectively] around each other. For the stimulation electrode, a distance between tips of 900 μm was maintained.

Depth electrodes were implanted stereotaxically (recording electrode: AP −5.3 mm, ML 3.2 mm relative to bregma; stimulation electrode: AP level of λ, ML 4.4 mm relative to bregma) according to the rat brain atlas (Paxinos and Watson, 2013). The exact dorsoventral position of the electrodes was based on electrophysiological feedback during surgery, so that the tips of the recording electrode were positioned in the subgranular layer of the DG upper blade. The stimulation electrode was positioned at the point where a maximal DG population spike was evoked.

All recorded signals were referenced to an epidural electrode above right frontal cortex. Recording and stimulation electrodes were connected to a headstage with a four-channel unity gain amplifier (based on TL074 SMT Opamp; Texas Instruments) through connector pins (W35520TRC, Winslow). A constant-current stimulator (DS4 Bi Phasic Stimulator; Digitimer) was used for electrical stimulation. EP sweeps were amplified 248 times, digitized at 20 kHz and high-pass filtered at 0.15 Hz. Digitization of signals was performed using a data acquisition device (USB-6259, 16-bit resolution, ± 10-V input range; National Instruments). Acquisition and offline analysis were performed using a MATLAB-based application (The MathWorks).

EPs were obtained in response to biphasic charge‐balanced square‐wave paired pulses delivered to the perforant path with a pulse width of 200 μs, intensities ranging from 25 to 1000 μA and an interstimulus interval of 10 s. The conditioning stimulus (EPa) and the test stimulus (EPb) were 10 ms apart. Stimulation intensity for both pulses was increased after every double pulse to obtain an input/output relationship. Per EP recording session, the set of intensities was repeated five times. One baseline recording sessions was performed, followed by subcutaneous injection of vehicle solution [1 ml/kg, 3.3% dimethyl sulfoxide (DMSO) in saline; Merck] and a second recording 10 min later. Clozapine (0.1 mg/kg in 0.3% DMSO in saline; Tocris Bioscience) was subcutaneously administered, followed by a 15-min waiting period and two more EP sessions, again with 10 min in between.

### Single-unit recordings

In four animals from the DM12 group, unit recording was performed under isoflurane anesthesia. Broadband (1–7500 Hz) electrophysiology signals were acquired at a sample rate of 30 kHz from the DG of the rat, using a 32-channel silicon probe (A1x32-Poly3-10 mm-50-177; Neuronexus), with the Open Ephys acquisition system (www.open-ephys.org) and an Intan amplifier/filter and digitizer boards (Intan Technologies). The Intan boards digitize the signals in a ± 6.4 mV, 16-bit integer range. The probe was lowered at in the right hippocampus 4.2 mm posterior and 3 mm lateral to λ. Activity was found around 3-mm depth and was estimated as putative DG activity. Around 30 min of baseline activity was recorded, followed by subcutaneous injection of vehicle and clozapine (0.1 mg/kg) and another ∼40 min of recording.

### Histology

Rats were killed immediately after EP recordings (*n*_DM13_ = 3, *n*_DM12_ = 8, *n*_DM11_ = 6, *n*_M13_ = 3) or at a later time point 2–23 weeks after the EP recording (*n*_D13_ = 8, *n*_DM13_ = 7, *n*_DM12_ = 1). Rats were deeply anaesthetized with an overdose of sodium pentobarbital (Dolethal, 200 mg/kg, i.p.; Vetoquinol) and transcardially perfused with PBS followed by 4% paraformaldehyde solution (Merck). The brains were removed and postfixed in paraformaldehyde solution for 24 h, cryoprotected in a 30% sucrose solution, frozen in ice-cold isopentane (Merck) and stored at −80°C until sectioning. Coronal sections (40 μm) were made at the level of the hippocampus using a cryostat (CM3050 S; Leica, RRID:SCR_016844).

In animals expressing mCherry (*n* = 27), immunofluorescence microscopy was performed on nine slices (ranging from −2.2 mm to −7.0 mm AP with an interslice distance of ∼0.6 mm according to the rat brain atlas; Paxinos and Watson, 2013) to evaluate mCherry expression. In all animals that were killed immediately after EP recordings (*n* = 17), an additional vimentin and CD11 b/c staining was performed on two separate slices at the level of the ventral injection site to assess the presence of vimentin^+^ reactive astrocytes (Ridet et al., 1997) and CD11b/c^+^ microglia or infiltrative neutrophils/monocytes (Jeong et al., 2013), respectively. Before incubation with primary antibody (rabbit anti-RFP, 1:1000; 600-401-379; Rockland/mouse anti-CD11b/c, 1:100; 550299; BD Biosciences/mouse anti-vimentin, 1:250; M072501-2; Dako), slices were blocked using a blocking buffer containing 0.2% Triton X-100 (Merck) and 0.4% fish skin gelatin (Merck). The next day, sections were incubated with secondary antibody (goat anti‐rabbit IgG Alexa Fluor 594, 1:1000; ab150088; Abcam/goat anti-mouse IgG Alexa Fluor 488; 1:1000; ab150113; Abcam) and a nuclear 4',6‐diamidino‐2‐fenylindool (DAPI) staining was performed (1 μg/ml; Merck). Slices were mounted and coverslipped with an antifade medium (Fluoroshield; Merck). Slides were digitized using a Pannoramic P250 Flash II scanner (3D-Histech) at 20× magnification using a DAPI and TRITC/FITC filter.

### Data analysis

Image analysis was performed using the image processing package Fiji, a distribution of ImageJ (http://ImageJ.net; Schindelin et al., 2012; Rueden et al., 2017). To determine the volume of right and left hippocampus, the intact hippocampus was delineated manually and its area was measured on eight consecutive T2-weighted MR images, ranging from −2.2 to −7.0 mm AP with an interslice distance of ∼0.6 mm (Paxinos and Watson, 2013). DG, cornu ammonis (CA) and subiculum were included in the delineated area. Hyperintensities and hypointensities were considered damage and excluded from the volume (Wolf et al., 2002). Total surface area of the granule and CA1 pyramidal cell layer were measured by delineating the cell layers on three DAPI-stained slices within a 2508 × 2508 μm region of interest (ROI) around the dorsal injection side. Intensities of mCherry, CD11 and vimentin staining were measured on their respective immunofluorescence images on one slice in four 250 × 250 μm ROIs at the level of the ventral injection side, both for the injected and non-injected hippocampus. mCherry intensities were also determined on an additional slice at the level of the dorsal injection side. All imaging and image analysis was conducted by investigators blinded to experimental conditions of the individual animals.

Analysis of EP data were performed blinded using MATLAB (v2018b; The MathWorks). One animal was excluded from the analysis, because of hardware problems during the recording (*n*_D13_ = 1). Three additional animals (*n*_DM12_ = 1, *n*_DM11_ = 2) were excluded because of insufficient DREADD-mCherry expression at the level of the recording electrode. Exclusion was based on visual assessment of the transduction as indicated by the anti-RFP staining, performed by researchers blinded to the experimental conditions. More specifically, the region at the level of the recording electrode was scored. A score between 0 (no expression) and 5 (very strong expression) was given for the granule cell layer and the hilus. The excluded animals all had a total score of 0. Slope of the field EPSP (fEPSP) was measured by fitting the rising phase of the fEPSP waveform using the least‐squares method. The area of the population spike was defined as the difference between the coastline of the negative peak of the population spike and the tangent connecting the positive peaks before and after the population spike. When multiple population spikes were present, areas were added up. For each animal and each parameter, the normalized values vehicle/baseline and clozapine/baseline were calculated at the stimulation intensity that during baseline recording resulted in a response with population spike area of 75% of the maximal response.

To extract multiunit activity, the broadband signals were bandpass filtered between 500 and 5000 Hz. Subsequently a thresholding method was used to detect spikes in SpykingCircus (v.1.0; https://spyking-circus.readthedocs.io/en/latest/). A threshold of −6.5 times the median absolute deviation of the signal was used. SpykingCircus was additionally used for spike sorting of single units. Subsequent manual curation of single unit clusters was done using the Phy GUI (https://phy.readthedocs.io/en/latest/visualization/). Firing frequency of multiunit or single-unit activity was computed in 60-s bins. Baseline and vehicle sessions were averaged and taken together as “baseline.”

The data analysis protocol was determined a priori based on previously obtained data. Based on a priori sample size calculations, minimal sample size per group was determined to be 5 animals to be able to detect a 10% difference on EP recordings with a power of 80%. Statistical analysis was performed in RStudio with R (v4.2; R core Team and R Foundation for Statistical Computing, 2017). Normality of the residuals was checked based on a qq plot and the Shapiro–Wilk test, homoscedasticity was visually checked and by means of the Levene’s test. Type III repeated-measures ANOVA was performed to detect effect of time on hippocampal volume, Type III ANOVA was performed to detect effect of injection or group on hippocampal volume, immunofluorescence staining intensities, layer thickness or fEPSP slope. Kruskal–Wallis rank sum test was performed to detect effect of group on population spike/fEPSP. Pairwise comparison was performed using two-tailed paired *t* tests, except for the unit data, on which a Wilcoxon signed rank test was performed. A Holm–Bonferroni correction was used for *post hoc* testing. Statistical significance was set at *p* < .05. Graphs were made using GraphPad Prism (v6.01; GraphPad Software).

## Results

### DREADD expression

**Figure 1.**
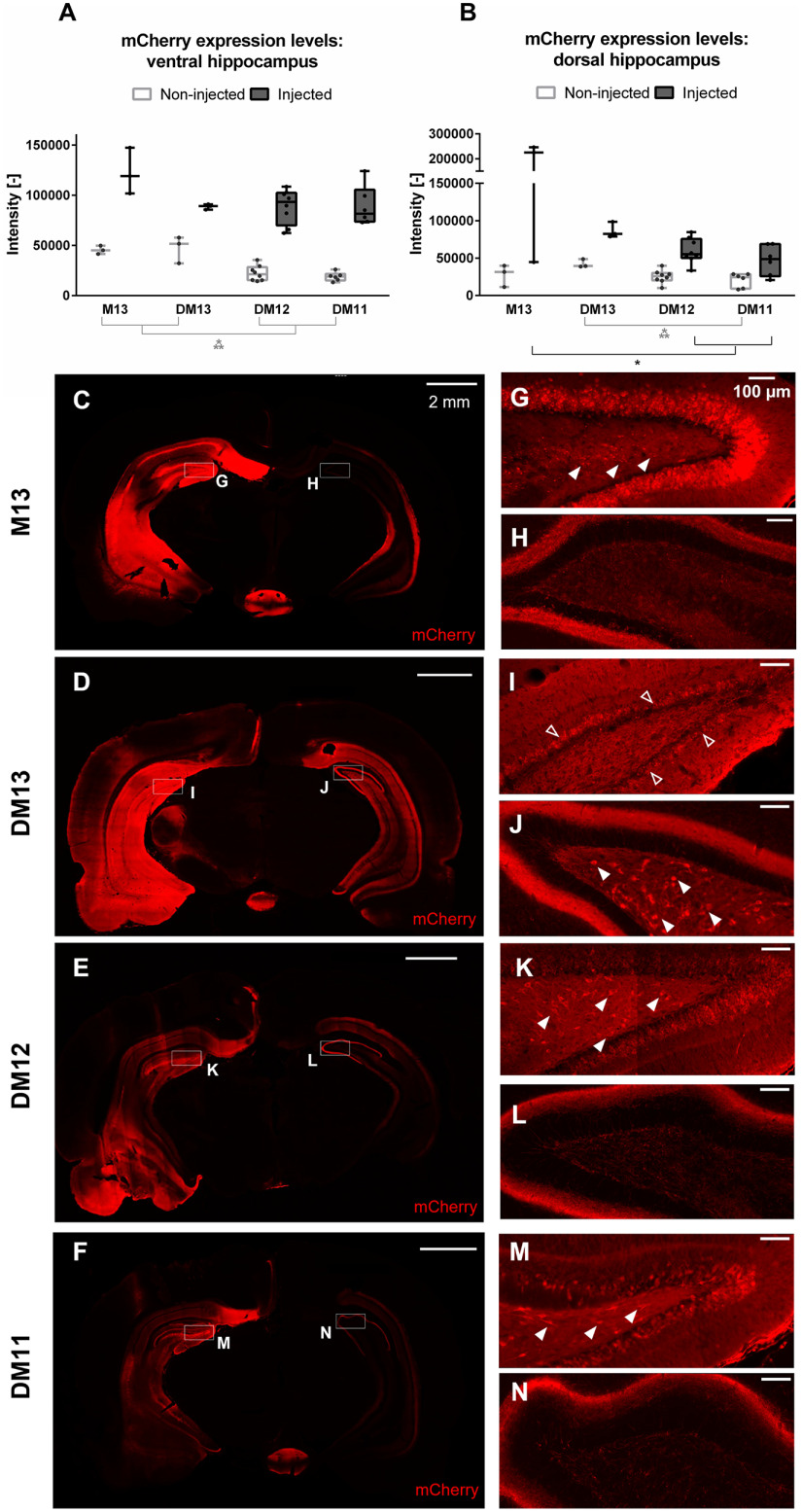
DREADD-mCherry/mCherry expression at the level of injection sites. ***A***, ***B***, Intensity of mCherry staining at the injected and non-injected hippocampus at the level of the ventral (***A***) and dorsal (***B***) injection site for different viral vector titers. A higher titer is linked to higher mCherry levels in both the injected and non-injected hemisphere. Dots represent individual animals. ***C–N***, Images at the level of the ventral injection site obtained from representative examples of the M13 (***C***), DM13 (***D***), DM12 (***E***), and DM11 (***F***) group, respectively, obtained five weeks after AAV injection. The injected hippocampus is situated on the left side. Boxes indicate zooms, corresponding with the hilus of the ipsilateral (***G***, ***I***, ***K***, ***M***) and contralateral (***H***, ***J***, ***L***, ***N***) DG. mCherry-positive hilar cells are indicated with filled arrowheads. On the DM13 image, the molecular layer of the DG is indicated with open arrowheads. On the injected side, both mCherry-positive cell bodies and fibers are present. On the non-injected side, mCherry expression is limited to fibers, except in the DM13 group, in which positive cell bodies are present too. Scale bars: 2 mm (***C–F***) and 100 μm (***G–N***). Exposure time: 20 ms for M13 and 25 ms for DM13, DM12, and DM11; *p < 0.05, ***p < 0.001. M = mCherry, DM = DREADD-mCherry. The high-resolution zooms were contrast-enhanced using the FIJI plugin “contrastenhancer” with 0.3% saturated pixels//normalize (unchecked)//equalize histogram (unchecked).

To evaluate the effect of viral vector titer on DREADD expression levels, different AAV titers were compared: E + 13, E + 12 and E + 11 vector genomes (vg)/ml (DM13, DM12, and DM11, respectively). A control group expressing unfused mCherry (M13; AAV titer of E + 13 vg/ml) was also included. Expression was visualized by an immunofluorescence staining against the mCherry tag. An example for each group obtained five weeks after AAV injection is given in Figure 1. Comparison between intensities of the mCherry tag in the injected and non-injected hippocampi (both ventral and dorsal) is given in Figure 1*A*,*B*. There was a significant interaction effect between titer and side on the expression levels of mCherry in the ventral (*F*_(3,16)_ = 3.30, *p* = 0.047) and dorsal (*F*_(3,16)_ = 8.27, *p* = 0.002) hippocampus. There was a significant effect of titer in the ventral (*F*_(3,16)_ = 16.67, *p* < 0.001) and dorsal (*F*_(3,16)_ = 3.54, *p* = 0.038) hippocampus on the non-injected side. Pairwise comparison showed that expression in the ventral hippocampus of DM13 and M13 was significantly different from expression in DM12 and DM11. For the dorsal hippocampus, expression in group DM13 was significantly different from DM11. At the injected side, there was a significant effect of group in the dorsal hippocampus (*F*_(3,16)_ = 6.49, *p* = 0.004). Pairwise comparison showed that expression in the M13 group was significantly different from the DM12 and DM11 group. Although in the injected ventral hippocampus higher intensities were observed with higher titers, effects were not statistically significant (*F*_(3,16)_ = 1.1, *p* = 0.362), probably because of the small sample size and the large variance within these groups. In the DM13 group, strong expression was observed throughout the entire injected hippocampus (Fig. 1*D*,*I*). Little to no hilar neurons were mCherry-positive on the ipsilateral side, whereas contralateral mCherry-positive hilar cells were observed in all animals of the DM13 group (Fig. 1*J*). In addition, positive cells were present in the CA2 and CA3 region of the contralateral hippocampus, as well as in the arcuate hypothalamic nucleus, medial amygdaloid nucleus and piriform cortex on the ipsilateral side. mCherry-positive fibers were also visible in cortical areas on the ipsilateral side including the retrosplenial cortex and piriform cortex, the mammillary bodies, fornix and the medial geniculate nucleus. In the animals injected with lower viral titers, expression patterns were more variable (Fig. 1*E*,*F*). The number and location of mCherry-positive cell bodies differed between animals within the same group. Strong granule cell and CA2/CA3 pyramidal cell expression was observed in most of the animals (DM12: 7/8; DM11: 4/6) on the injected side, although expression was less pronounced than in the DM13 group. mCherry-positive hilar cells were present in part of the animals (DM12: 5/8; DM11: 3/6; Fig. 1*K*,*M*). mCherry-positive fibers were also observed in the contralateral hippocampus (Fig. 1*L*,*N*) and cortical areas on the ipsilateral side including the retrosplenial and piriform cortex and the mammillary bodies. Compared with the DM13 group, cortical expression was limited. No mCherry-positive cell bodies were detected in the contralateral cortex. In some animals, mCherry-positive cortical cells were present around the injection tracts. Two animals of the DM11 group and one animal of the DM12 group displayed very low DREADD-mCherry expression at the level of the recording electrode and were excluded from electrophysiological analysis. In the mCherry control group, the strongest mCherry expression was observed, reflected by very high intensities despite a lower exposure time (Fig. 1*C*). mCherry-positive cells were situated in both cell bodies and fibers of the entire injected hippocampus (Fig. 1*G*), in cell bodies of the ipsilateral amygdaloid nuclei and striatum and in fibers of the ipsilateral retrosplenial cortex and fornix, mammillary bodies and the contralateral hippocampus (Fig. 1*H*). Apart from the retrosplenial cortex, no cortical mCherry expression could be observed. There was no evidence for transduction to non-neuronal cells in any of the groups, although this was not confirmed by a neuronal staining.

### Neurotoxicity

**Figure 2.**
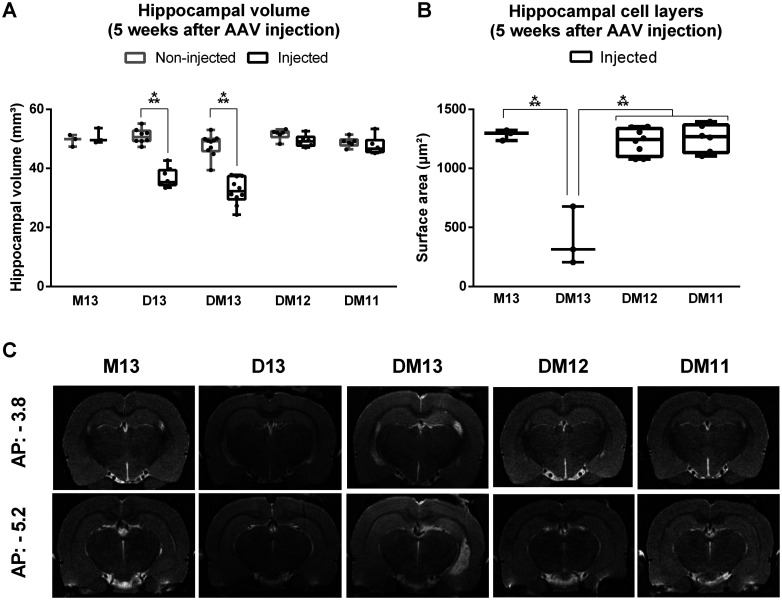
Effect of DREADD expression level on hippocampal volume and cell layers at five weeks after AAV injection. ***A***, Volume of the injected hippocampus is significantly reduced compared with the non-injected hippocampus in the DREADD and DREADD-mCherry group with titer E + 13 vg/ml. ***B***, Thickness of the DG and CA1 hippocampal cell layers is significantly reduced in the injected hippocampus in the DREADD-mCherry group with titer E + 13 vg/ml compared with the other groups. ***C***, Representative example of T2 MRI scans at the anterior and posterior injection sites for different groups. The injected hippocampus is situated in the MR images on the right side; ***p < 0.001. Dots represent individual animals. AP = anteroposterior, M = mCherry, D = DREADD, DM = DREADD-mCherry.

To evaluate the effect of viral vector titer and subsequent DREADD expression levels on neuronal death, the volumes of injected and non-injected hippocampi (Fig. 2*A*,*C*) and thickness of the granule cell layer and CA1 pyramidal cell layer on the injected side (Fig. 2*B*) were compared at five weeks after transduction. Different titers (E + 13, E + 12 and E + 11 vg/ml) reflecting different levels of expression were compared (DM13/D13/M13, DM12, and DM11, respectively). To assess the effect of the expressed protein, unlabeled DREADD (D13), DREADD fused to mCherry (DM13), and unbound mCherry (M13) were compared within the highest titer condition. There was a significant interaction effect between group and side on hippocampal volume (*F*_(4,28)_ = 33.47, *p* < 0.001). There was a significant effect of group on the volume of the injected hippocampus (*F*_(4,28)_ = 38.47, *p* < 0.001), but not on the volume of the non-injected hippocampus (*F*_(4,28)_ = 2.43, *p* = 0.071). Pairwise comparison showed that volume of the injected hippocampus was significantly lower compared with the non-injected hippocampus in the DM13 (*t* = 9.92, df = 9, *p* < 0.001, mean difference of −15.3 ± 3.5 mm³) and D13 (*t* = 12.02, df = 7, *p* < 0.001, mean difference of −14.1 ± 2.8 mm³) group. There was a significant effect of group on the thickness of the cell layers on the injected side (*F*_(3,16)_ = 35.34, *p* < 0.001). Pairwise comparison showed that layer thickness was significantly lower in the DM13 group compared with the M13, DM12, and DM11 group (*t*_DM13-M13_ = 8.23, *p* < 0.001; *t*_DM13-DM12_ = 9.27, *p* < 0.001; *t*_DM13-DM11_ = 9.19, *p* < 0.001; df = 16; mean difference of −847 μm^2^).

**Figure 3.**
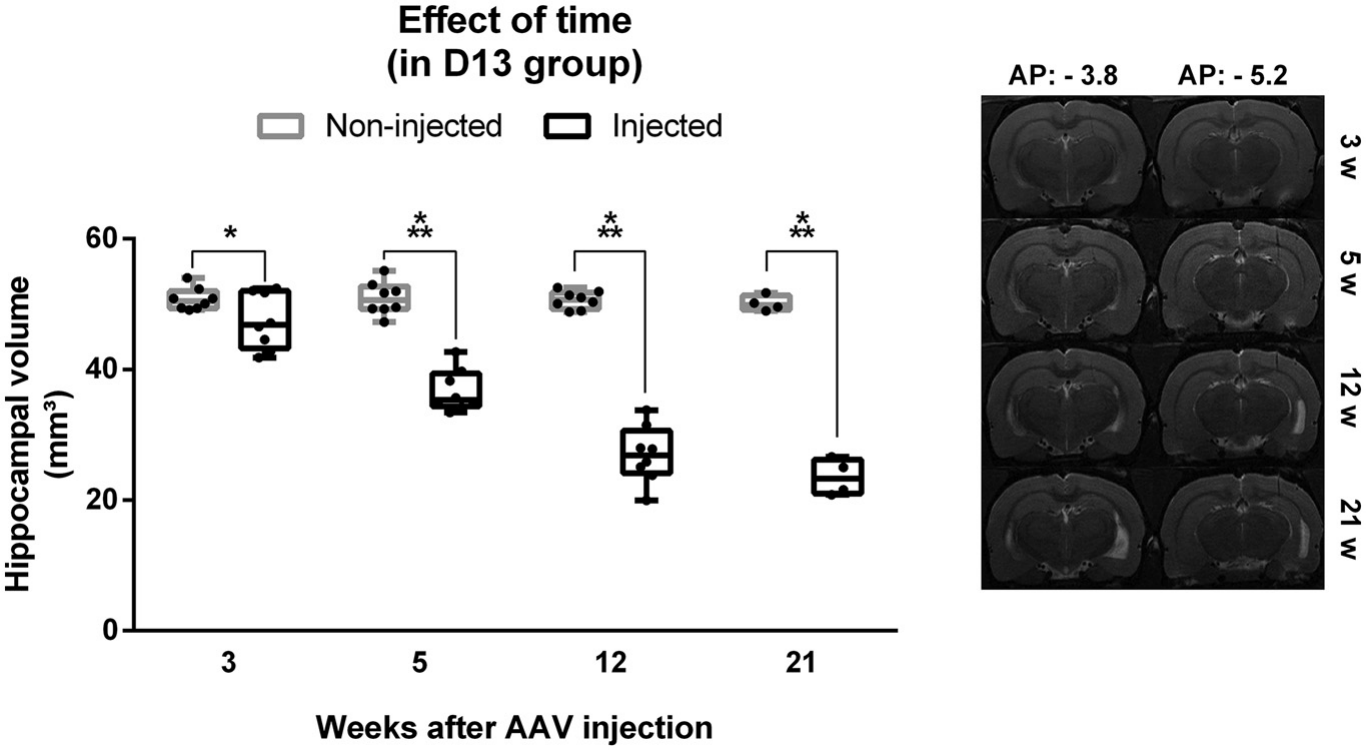
Effect of time on hippocampal volume in the DREADD group with titer E + 13 (D13). Left, Volume of the injected hippocampus is significantly reduced compared with the non-injected hippocampus at all time points and the difference in hippocampal volume between the injected and non-injected hippocampus increases with time. Dots represent individual animals. Right, Representative example of T2 MRI scans at the anterior and posterior injection sites at different timepoints in one animal. The injected hippocampus is situated in the MR images on the right side; *p < 0.05, ***p < 0.001. AP = anteroposterior.

To map the development of neuronal loss over time, anatomic T2-weighted MRI scans were performed 3, 5, 12 (*n*_D13_ = 8), and 21 (*n*_D13_ = 4) weeks after viral vector transduction in a longitudinal study in the unlabeled DREADD group (D13). The volume of the injected and non-injected hippocampi were determined at different time points after injection (Fig. 3). There was a significant interaction effect between time after AAV injection and side on hippocampal volume (*F*_(3,41)_ = 63.39, *p* < 0.001). Statistical analysis showed a significant effect of time in the injected hippocampus (*F*_(3,9)_ = 147.64, *p* < 0.001) but not in the non-injected hippocampus (*F*_(3,9)_ = 0.97, *p* = 0.447). Pairwise comparison for each time point showed that hippocampal volume was significantly lower for the injected hippocampus compared with the non-injected hippocampus at all time points (3 weeks: *t* = 3.2604, df = 7, *p* = 0.014, mean difference of −3.4 ± 2.4 mm³; 5 weeks: *t* = 12.016, df = 7, *p* < 0.001, mean difference of −14.2 ± 2.8 mm³; 12 weeks: *t* = 17.169, df = 7, *p* < 0.001, mean difference of −23.6 ± 3.3 mm³; 21 weeks: *t* = 15.946, df = 3, *p* = 0.001, mean difference of −26.6 ± 5.3 mm³). There was a significant decrease in hippocampal volume of the injected hippocampus between week 3 and week 5 (*p* = 0.023, *t* = 12.95, df = 7, mean difference of −10.7 ± 3.3 mm³), week 5 and week 12 (*p* = 0.022, *t* = 14.11, df = 7, mean difference of −9.7 ± 2.9 mm³), and week 12 and week 21 (*p* = 0.030, *t* = 3.89, df = 3, mean difference of −4.1 ± 3.3 mm³).

**Figure 4.**
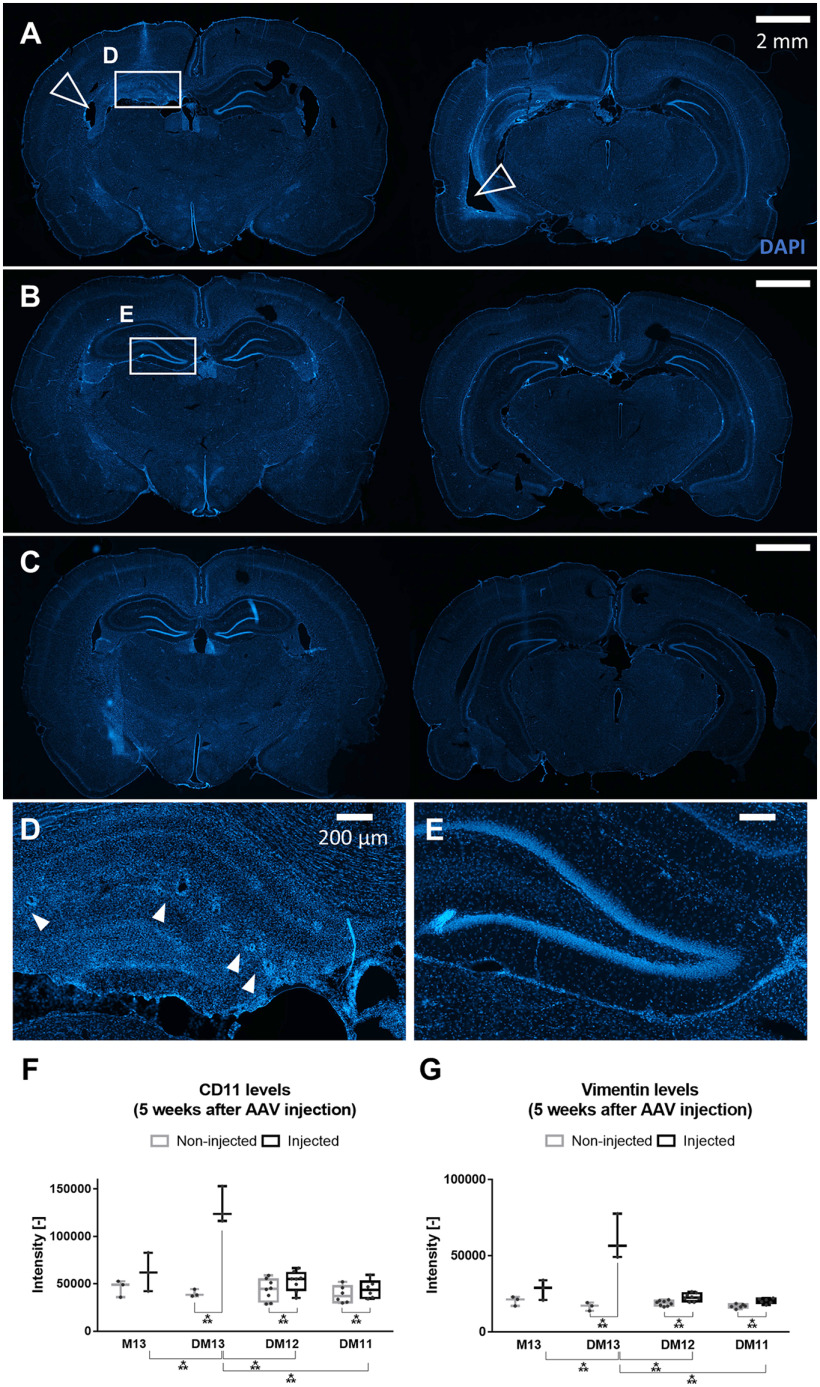
Histologic analysis shows profound hippocampal degeneration, atrophy, and inflammation in the injected hemisphere of the DM13 group. Representative nuclear (DAPI) staining images of an animal from the DM13 (***A***, ***D***), M13 (***B***, ***E***), and DM12 (***C***) group at the level of the dorsal and ventral injection site, five weeks after AAV injection. The injected hippocampus is situated on the left side in the images. Hippocampal degeneration is observed in the injected side (***D***) in the DM13 animal, but not in non-injected hippocampus or the injected hippocampus of an animal from the control mCherry group (***E***). Filled arrowheads indicate holes, presumably caused by enlarged blood vessels. Blank arrowheads indicate enlarged ventricles. Scale bars: 2 mm (***A–C***) and 200 μm (***D***, ***E***). Exposure time: 20 ms. ***F***, ***G***, CD11 (***F***) and vimentin (***G***) levels in the different groups at five weeks after AAV injection. CD11 and vimentin are slightly upregulated in the injected hippocampus of all groups, presumably because of the physical injection itself. Both inflammatory markers are significantly more upregulated in the injected hippocampus of the DM13 group compared with the other groups. Dots represent individual animals; ***p < 0.001.

Sections obtained five weeks after AAV injection of DREADD-mCherry (DM13, DM12, DM11) and mCherry (M13) expressing animals were stained for the presence of microglia or infiltrative monocytes/neutrophils (CD11; Fig. 4*F*) and reactive astrocytes (vimentin; Fig. 4*G*), alongside a nuclear staining (DAPI; Fig. 4*A–E*). The nuclear staining revealed profound hippocampal degeneration and atrophy and enlarged lateral ventricles in the injected hemisphere of the DM13 group, compared with the non-injected side (Fig. 4*A*,*D*). Neuronal loss in all hippocampal cell layers was observed, as well as vacuolation of the tissue, presumably caused by enlarged blood vessels. No such effects were observed in animals from the mCherry control group (Fig. 4*B*,*E*), nor in animals of the lower titer group DM12 (Fig. 4*C*). Statistical analysis showed a significant interaction effect between group and side on CD11 (*F*_(3,16)_ = 85.24, *p* < 0.001) and vimentin (*F*_(3,16)_ = 55.93, *p* < 0.001) intensities. There was a significant effect of group for the CD11 and vimentin intensities in the injected side (*F*_(3,16)_ = 32.08, *p* < 0.001 and *F*_(3,16)_ = 35.10, *p* < 0.001, respectively), but not in the non-injected side (*F*_(3,16)_ = 0.53, *p* = 0.67 and *F*_(3,16)_ = 2.93, *p* = 0.06, respectively). Pairwise comparison showed that intensities on the injected side in the DM13 group were significantly different from intensities in the other groups for both markers. The difference between injected and non-injected side was significant in each DREADD-mCherry group for CD11 (*t*_DM13_ = −10.22, *p*_DM13_ = 0.019; *t*_DM12_ = −5.52, *p*_DM12_ = 0.003; *t*_DM11_ = −9.07, *p*_DM11_ = 0.001) and vimentin (*t*_DM13_ = −6.24, *p*_DM13_ = 0.049; *t*_DM12_ = −5.03, *p*_DM12_ = 0.006; *t*_DM11_ = −5.42, *p*_DM11_ = 0.009), but larger in the DM13 condition compared with the other groups (CD11: mean difference of −91,185 vs −5519 and −5573; vimentin: mean difference of −44,322 vs −3993 and −3396).

### Neurophysiological effects of DREADD activation

#### Effect on DG evoked potentials

To evaluate the electrophysiological effects of DREADD-mediated inhibition of hippocampal excitatory neurons, DG EPs were recorded. DG EPs are field potentials evoked by activation of the perforant path, a bundle of axons projecting from the entorhinal cortex to the DG. The EPs have a characteristic pattern composed of a fEPSP, representing glutamatergic neurotransmission between perforant path and DG granule cells, and population spike(s) (PS), reflecting summed action potentials of the granule cells (Sloviter, 1991). Activation of the inhibitory hM4D(Gi) receptor in the hippocampal granule cells is hypothesized to suppress synaptic transmission, as reflected by a decrease in all EP parameters.

**Figure 5.**
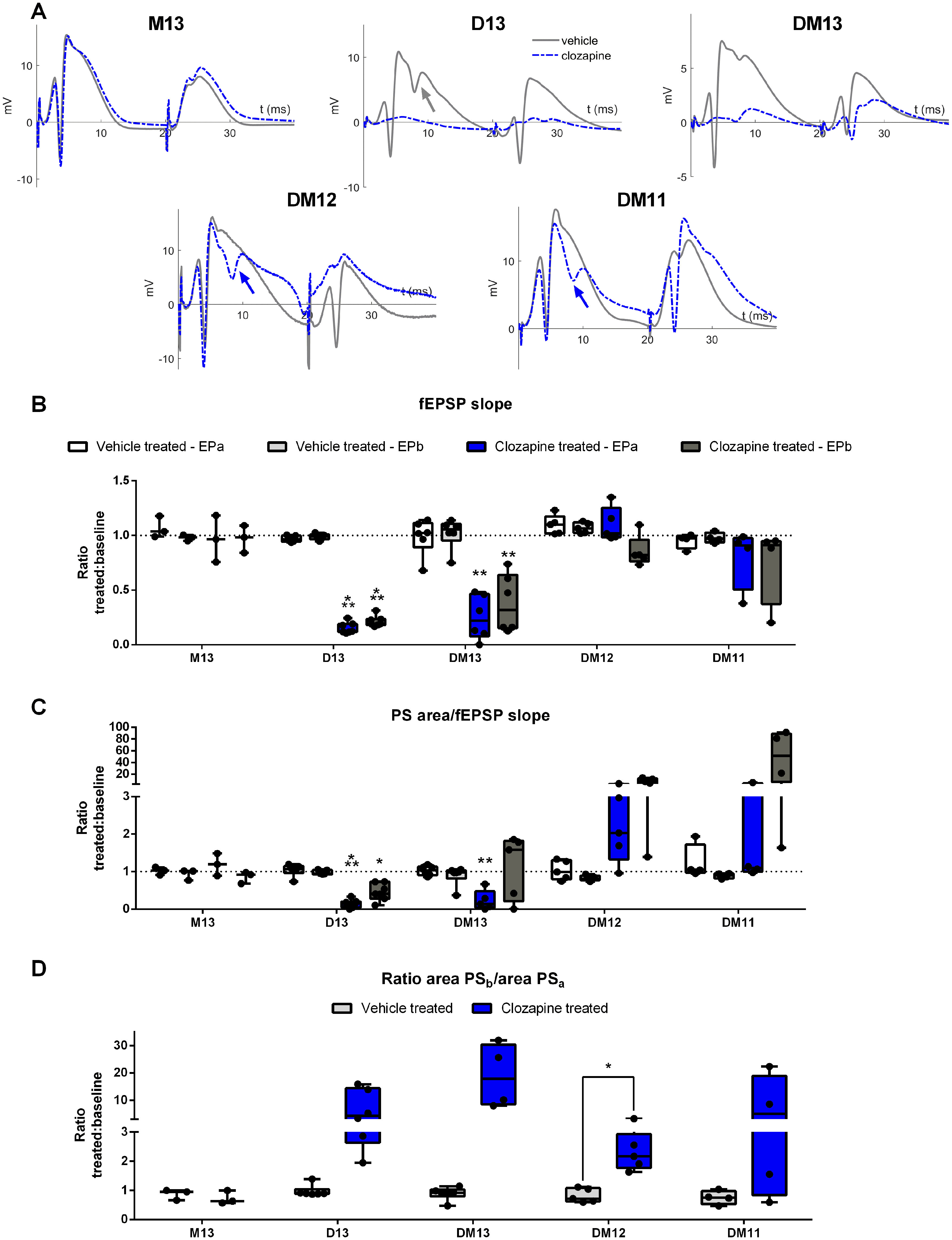
Effect of clozapine-mediated hM4D(Gi) activation on DG evoked potentials. ***A***, Representative EP traces obtained during vehicle (gray) and clozapine (blue) treatment. Multiple population spikes are indicated with arrow. ***B***, fEPSP slope was significantly reduced in the D13 group and DM13 group with titer E + 13 vg/ml, both for the first EP (EPa) and the second EP (EPb) compared with vehicle injection. ***C***, Population spike area/fEPSP slope ratio was significantly reduced in the D13 group and the DM13 group for EPa, and for the D13 group for EPb, compared with vehicle. In some animals from the DM12 and DM11 group with lower titers, population spike/fEPSP slope ratio was strongly increased, although not significantly. ***D***, Paired pulse relationship was increased during clozapine treatment compared with vehicle for all DREADD groups, but effects were only significant in the DM12 group. Dots represent individual animals; *p < 0.05, **p < 0.01, ***p < 0.001. D = DREADD, M = mCherry, DM = DREADD-mCherry, PS = population spike, fEPSP = field EPSP.

DG EPs were recorded before and after clozapine administration. Compared with baseline, clozapine had a group-dependent effect on fEPSP slope of both the first (EPa) and the second EP (EPb; EPa: *F*_(4,23)_ = 40.02, *p* < 0.001; EPb: *F*_(4,23)_ = 16.16, *p* < 0.001; Fig. 5*B*) and the population spike area/fEPSP slope ratio of both EPs (EPa: χ^2^ = 14.65, df = 4, *p* = 0.005; EPb: χ^2^ = 16.83, df = 4, *p* = 0.002; Fig. 5*C*). fEPSP slope of both EPs was significantly reduced in the D13 (EPa: *t* = −151.77, df = 5, *p* < 0.001, mean difference of −83 ± 2%; EPb *t* = −36.21, df = 5, *p* < 0.001, mean difference of −77 ± 5%) and the DM13 group (EPa: *t* = −8.21, df = 5, *p* = 0.002, mean difference of −74% ± 14; EPb: *t* = −6.37, df = 5, *p* = 0.006, mean difference of −64 ± 25%). Population spike area/fEPSP slope ratio was significantly reduced for EPa and EPb in the D13 group (EPa: *t* = −15.17, df = 5, *p* < 0.001, mean difference of −87 ± 15%; EPb: *t* = −5.64, df = 5, *p* = 0.012, mean difference of −46 ± 21%) and for EPa in the DM13 group (*t* = −8.13, df = 4, *p* = 0.005, mean difference of −80 ± 28%) compared with the vehicle-treated condition. In the other groups (M13, DM12, and DM11), no significant differences between vehicle and clozapine were observed for fEPSP slope or population spike/fEPSP slope ratio. However, in five out of eight animals of the DM12 group and two out of four animals of the DM11 group, multiple population spikes were observed following hM4D(Gi) activation (Fig. 5*A*, indicated with arrows). The excitatory effects were also reflected in an increase in PS/fEPSP slope ratio (i.e., intrinsic excitability), although not significant, likely because of the small sample size and the large variability (Fig. 5*C*). In all animals of the DM12 and DM11 group, a broadening of the fEPSP was observed. In all DREADD-expressing animals, paired-pulse inhibition was reduced following clozapine administration, reflected by a higher EPb/EPa ratio (Fig. 5*D*), although only significant differences between vehicle and clozapine were observed in the DM12 group (*t* = 4.53, df = 5, *p* = 0.03, mean difference of 134 ± 76%). In the high titer groups, increased EPb/EPa ratio is partly caused by suppression of the first pulse EPa. In the lower titer groups, the increased paired-pulse relationship is solely caused by an increase in EPb population spike. In four out of ten animals of the DM13 group, EPs could not be recorded despite adequate electrode placement, likely because of extensive hippocampal degeneration as revealed by MRI and postmortem histologic examination (Figs. 2, 4).

#### Effect on spontaneous neuronal activity

**Figure 6.**
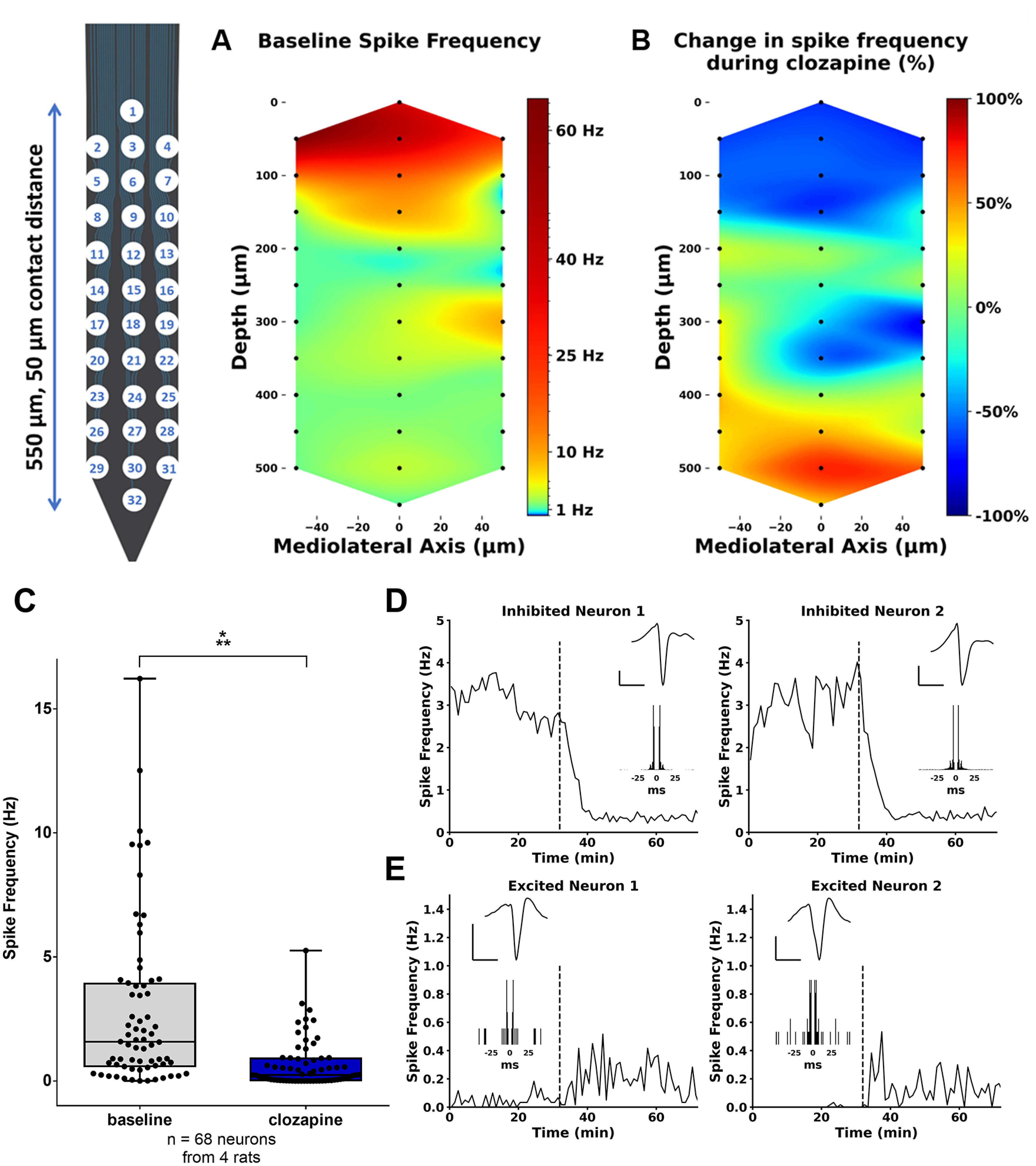
Effect of clozapine-mediated hM4D(Gi) activation on unit activity in the DG. ***A***, Graphical overview of the average multiunit firing frequency across the probe during the baseline period. The baseline firing frequency was around 50–60 Hz on the channel displaying the most activity (channel 2), while less activity was observed on the majority of the contacts of the probe. ***B***, Graphical overview of relative change in firing frequency across the probe after clozapine administration, displaying a decrease in spike frequency in the upper channels and an increase in the lowest channels. ***C***, Spike frequency for all isolated neurons during baseline and after clozapine administration. Data represented as median and interquartile range. Dots correspond to individual neurons. ***p < 0.001. ***D***, ***E***, Representative examples of single units and their firing rates before and after clozapine administration, as indicated by the dashed line. Average action potential waveforms on the peak channels and auto-correlogram are displayed in the insets. Units located on the dorsal channels of the probe were inhibited following clozapine injection (***D***). Units located on the ventral channels of the probe had a tendency to increase their firing following clozapine administration (***E***).

To examine the effect of DREADD activation on spontaneous firing, unit recordings were performed in four animals of the DM12 group that displayed a clear excitatory effect on EPs following DREADD activation (Fig. 5*A*, DM12). Unit activity was mainly detected in the dorsal channels of the probe, which was placed at the dorsoventral coordinates of the upper DG blade (Fig. 6*A*). In these channels, multiunit spike activity was strongly reduced after clozapine administration (Fig. 6*B*). In the ventral channels of the probe that were presumably located in the hilus of the DG, a relative increase in multiunit spike frequency was observed (Fig. 6*B*). Isolation of single-unit activity reflected the observation made at a multiunit level, showing a significant decrease in firing frequency on clozapine administration compared with baseline (*W* = −1650, *p* < 0.001, median difference of −1.07; Fig. 6*C*). All of the inhibited units were located in the most dorsal channels of the probe (Fig. 6*D*). A few units in the most ventral channels showed increased firing rates following clozapine administration (Fig. 6*E*).

## Discussion

### Neurotoxic effects

Our results indicate that local cell death progressively develops following AAV vector injection and subsequent DREADD expression in the hippocampus. At the three weeks after transduction time point, a minor decrease in hippocampal volume could be observed. However, at later timepoints the decrease in hippocampal volume became more prominent, resulting in hippocampal volumes of only ∼50% of their initial value. In addition, the lateral ventricle was increased and T2-weighted hyperintensities were visible within the hippocampus. Histologic analysis demonstrated neuronal loss, degeneration of the hippocampal cell layers, inflammation and vacuolation of the tissue. Although the detrimental effect aggravates progressively, the largest changes seem to occur during the first 12 weeks. All experiments were performed in male rats to ensure homogeneity in brain sizes.

We found that occurrence of hippocampal cell loss is highly dependent on the DREADD expression level, determined in the experiments by a varying viral titer of the AAV. DREADD groups displaying a high level of expression (AAV vector titers of E + 13 vg/ml) displayed clear hippocampal lesions and elevated levels of microglia or infiltrative monocytes/neutrophils and reactive astrocytes in the injected hippocampus, whereas in DREADD groups displaying less pronounced expression (dilution of the AAV vector titer to E + 12/E + 11 vg/ml) no significant reduction in hippocampal volume was observed. Apart from a minor increase in inflammatory markers, presumably caused by the physical injection itself, inflammation was not observed in these groups. No deleterious effects were observed five weeks after transduction in the brains of animals injected with the mCherry control vector either, despite strong mCherry expression (AAV vector titer of E + 13 vg/ml). The occurrence of neuronal loss was not influenced by the tag fused to the DREADD, since there were no significant differences between conditions with matched titer and volume but without molecular tag. Overall, our results indicate a toxic effect in the hippocampus specifically caused by high levels of expression of the DREADD hM4D(Gi), but not by expression of other proteins nor by introduction of the viral vector itself (e.g., capsid).

Differences in neurotoxicity (i.e., tissue damage, neuronal loss, and inflammation) could be explained by the membrane-associated nature of the hM4D(Gi) receptor. In microorganisms, overexpression of membrane proteins is frequently associated with high levels of toxicity (Wagner et al., 2007). In mammalian cell lines, slow growth is associated with using strong constitutive promoters for the production of membrane proteins, likely because of the high metabolic demands placed on the cells or on the adverse biological activity of the membrane protein itself (Andréll and Tate, 2013). High-level transgene overexpression can lead to an overload of the protein biosynthetic machinery, the chaperones responsible for protein folding or the translocon responsible for transport and integration of proteins to the cell membrane (Kintaka et al., 2016). Accumulation of unfolded proteins in the endoplasmic reticulum (ER), results in ER stress and triggers a series of signaling events to restore normal function. However, when the disruption is prolonged, apoptosis will be induced (Almanza et al., 2019; Kim et al., 2008; Tabas and Ron, 2011). In addition, accumulation of non-native structures in a membrane can induce stress responses in the host (Gialama et al., 2017). The expression of a human protein in another species can also elicit an immune response toward the foreign protein.

Since only for the strongest DREADD-expressing condition, neuronal loss is observed already at early timepoints after transduction, this could explain why DREADD-mediated toxicity following AAV injection remains unreported. Only few rodent studies used equally high viral vector titers in combination with relatively large injected volumes, resulting in similar expression profiles. Furthermore, part of these studies only looked at the outcome at three to four weeks after transduction (Ferguson et al., 2013; Grace et al., 2018; Maharjan et al., 2018; Hu et al., 2019). Although we observed some minor cell loss at three weeks after transduction, the effects were subtle and could be easily overlooked. No reports are available on long-term DREADD expression specifically in CaMKIIα-positive excitatory neurons in rodents. In rodent studies using different promoters and AAV serotypes but similar viral titers and injected volumes, DREADDs were expressed in the hippocampus (Panoz-Brown et al., 2018) or in a different brain region such as the retrosplenial cortex (Robinson et al., 2014), locus coeruleus (Rorabaugh et al., 2017), or striatum (Aldrin-Kirk et al., 2016). In these studies, no adverse effects were reported 3 (Robinson et al., 2014; Panoz-Brown et al., 2018), 6 (Rorabaugh et al., 2017), or 18 weeks (Aldrin-Kirk et al., 2016) after viral transduction. In non-human primate studies, long-term experiments are more common. No reports are available on DREADD-induced toxicity in these studies either (Gray et al., 2011; Eldridge et al., 2016; Galvan et al., 2019; Nagai et al., 2020; Upright and Baxter, 2020). It should however be noted that the affinity of an AAV serotype depends on brain region and species, which can affect to the level of DREADD expression (Howard et al., 2008). In addition, viral titer quantification using qPCR is only indicative, and comparing vector titers between different research groups is difficult. Therefore, a similar titer is no guarantee for similar transduction levels (Ayuso et al., 2014). This could also imply that the titers we used are actually higher, or that viral titers were overestimated in other studies. In addition, a titer expressed in viral genomic copies does not hold any information of the number of infectious particles, nor on the number of empty viral capsids. A large number of empty capsids could also contribute to toxicity (Hinderer et al., 2018).

There are some reports on neuronal loss and degeneration of glia induced by AAV-mediated overexpression of other proteins in *in vivo* studies, that did not occur in control conditions where green fluorescent protein was expressed (Golebiowski et al., 2017; Amado et al., 2019). One study reports toxicity when hM3(Dq) is expressed in the locus coeruleus of rats following CAV-mediated transduction (Stevens et al., 2020). Neurotoxicity, however, has been reported with green fluorescent protein expression too when high titers were used, even with subretinal administration of AAV, which has applications that are clinically approved (Howard et al., 2008; Samaranch et al., 2014; Khabou et al., 2018; Xiong et al., 2019). The fact that similar toxicities were observed with different transgenes and viral capsids and in different brain regions, suggests that this may represent a property of the viral vector platform rather than responses to specific proteins or vector constructs. These findings underscore the need for careful control of transgene overexpression-induced toxicity in the brain, especially when investigating the role of specific proteins in pathologies characterized by neuronal degeneration.

### Neuromodulatory effects

To assess the neurophysiological effects of DREADD activation on the hippocampal cells, DG evoked potentials following paired-pulse perforant path stimulation were recorded. In the groups displaying strong hM4D(Gi) expression following high titer AAV injection, strong inhibitory effects were observed after clozapine-mediated DREADD activation. Excitatory synaptic transmission was significantly decreased, as reflected by the slope of the fEPSP. In animals where a minimal fEPSP could still be recorded after clozapine administration, the intrinsic excitability was significantly suppressed. No significant effects were observed in the mCherry control group, indicating that the observed effects are indeed the result of hM4D(Gi) activation. These findings are consistent with previous reports on the use of hM4D(Gi) (Armbruster et al., 2007; Stachniak et al., 2014).

No significant inhibitory effects on EP activity were observed for the groups injected with a lower titer AAV, resulting in more moderate, non-toxic hM4D(Gi) expression. In contrast, a broader fEPSP, larger, and more population spikes and reduced paired-pulse inhibition were seen in the majority of these animals. When looking at a neuronal level, in the dentate granule cell layer spontaneous firing of all neurons was inhibited following DREADD activation. In contrast, a few neurons located deeper in the hilus of the DG (∼400 μm ventral to the dentate granule cell layer) showed an increase in spontaneous firing following clozapine administration. It should, however, be noted that we were not able to distinguish granule cells from interneurons or mossy cells in this limited data set. Nevertheless, the excitatory effects seem paradoxical, as hM4D(Gi) is widely used as a tool to silence neurons (Zhu and Roth, 2014). What exactly causes the discrepancy between the effect observed with the highest titer and the lower titers and between the unit and EP recordings, is uncertain.

On the one hand, effects could be directly related to differences in titer, resulting in different DREADD expression patterns. There are some reports on similar mixed effects on unit activity following chemogenetic interventions. These effects are hypothesized to be the result of disruption of neuronal activity in a target area, involving disinhibition of some DREADD-negative cells as a result of changes in local microcircuitry or wider networks (Vazey and Aston-Jones, 2014; Chang et al., 2015). Although hM4D(Gi) is thought to reduce neuronal firing through hyperpolarization of the cell, this effect might be limited when lower numbers of receptors are expressed as in the case with the lower titers in groups DM12 and DM11 (Armbruster et al., 2007). Especially with evoked neuronal activity, a moderate hyperpolarizing effect might not be sufficient to prevent action potentials. When the main effect of hM4D(Gi) activation consists of blockage of neurotransmitter release, granule cells in the lower titer conditions will still be activated, but without passing on signals to neighboring cells. As the DG circuitry is characterized by a high prevalence of lateral inhibition, this could eventually result in reduction of interneuron activity and disinhibition of the granule cells (Espinoza et al., 2018). Loss of feedback inhibition is consistent with the distorted fEPSPs characterized by an increased paired-pulse relationship and the multiple granule cell population spikes observed after clozapine treatment in the groups DM12 and DM11 (Sloviter, 1991). In higher titer conditions the situation could be different, since more extrahippocampal DREADD expression was seen in this group. When afferent regions of the DG are transduced as well, e.g., the entorhinal cortex, synaptic input to the granule cells will be reduced, contributing to an inhibitory effect. We were not able to confirm or disprove this hypothesis based on histologic analysis, as we did not include the perforant path in our staining. However, immunofluorescence staining at the level of the recording electrode showed stronger intensities in the high titer condition compared with low titer conditions for the molecular layer of the DG, where the fibers of the perforant path innervate the granule cells. In addition, fEPSP slope was reduced in the high titer conditions. Although this could also have been caused by shunting inhibition, it is plausible that DREADD expression in the entorhinal cortex was responsible for this effect.

Alternatively, effects can be indirectly linked to titer, because high DREADD expression levels clearly result in hippocampal neurotoxicity, which possibly creates a pathologic environment. Loss of interneurons for example would already disturb the feedback inhibition circuits even without DREADD activation. In addition, mossy cells, highly active glutamatergic neurons that indirectly exert a net inhibitory effect on neighboring granule cells, are also known to be particularly sensitive to toxicity (Jinde et al., 2012; Scharfman, 2018). Subsequent changes in plasticity will create an entirely different situation, possibly explaining the discrepancy in effects between the different titers.

Our findings indicate that level of DREADD expression plays an important role on the final outcome of hM4D(Gi) activation, at least for evoked activity. Further research in a larger group of animals, both at single neuron and field potential level, is required to elucidate the precise mechanism of action.

We conclude that the titer of the viral vector used to obtain DREADD expression plays a role in the development of hippocampal neurotoxicity, as well as in the expression pattern, which in turn seem to affect the net modulatory effects following DREADD activation. This has possible implications for all DREADD-based research. Based on this study we would recommend to be cautious for deleterious effects of DREADD expression to avoid confounding of results and mention the titer of the AAV used. Especially with regard to therapeutic applications of DREADDs, long-term studies on toxicity are advised. In addition, DREADD function should be assessed electrophysiologically, before inferring conclusions from, e.g., behavioral studies, as DREADD-mediated effects might be dependent on expression pattern.

## Synthesis

Reviewing Editor: Deanna Smith, University of South Carolina Decisions are customarily a result of the Reviewing Editor and the peer reviewers coming together and discussing their recommendations until a consensus is reached. When revisions are invited, a fact-based synthesis statement explaining their decision and outlining what is needed to prepare a revision will be listed below. The following reviewer(s) agreed to reveal their identity: NONE.

The reviewers indicated that this is a very interesting and timely set of studies, dealing with aspects that are usually overlooked in chemogenetic experiments. The question is important, the methodology is appropriate. The study will help move the field forward in that it provides an important cautionary tale with regards to an increasingly common methodology in the field. However, both reviewers had some concerns that need to be addressed. Their comments are included in full below:

Reviewer 1:

Major

1. One main concern is regarding the statements of neuronal cell loss. This statement is based, as far as I could understand, on the MRI images. Although the atrophy and loss of volume is obvious, a definitive statement about loss of neuronal cells should be based on stereological counts on tissue stained for neuronal markers (such as NeuN). Without this evidence, I suggest toning down the description of cell loss. The evidence shown with the MRI is indicative of tissue damage.

2. On a related note, clarify what is neurotoxicity in the context of this study. Does this refer to neuronal death? If neurotoxicity is evaluated only on the basis of the volume of the hippocampi, there should be some description on how the volume of the structure assessed by MRI reflects neurotoxicity.

3. To solidify the statement about neuronal degeneration, the manuscript should include experiments with markers of neuronal cell death or degeneration. Markers of inflammatory processes could also be used.

4. Another important concern is that the comparisons of levels of transduction with DM13, DM12 and DM11 are only descriptive. Although the examples shown in figure 1 indicate a stronger transduction for the animal in the DM13 group, the question remains how much stronger this transduction is compared to DM12 and DM11. Can these differences in expression be quantified?

5. Volumes injected per site (3 ul) are high. It is likely that each of these diffused to areas covered, at least in part, by the other injection. Can the 2 injections be identified separately?

6. Clarify in manuscript if the recordings of single units described starting page 7 done in anesthetized animals, as described for the EP recordings.

7. Page 8: “Three additional animals (nDM12 = 1, nDM11 = 2) were excluded due to insufficient DREADD-mCherry expression as confirmed on histological analysis.” What was considered ‘sufficient’ expression?

8. Group “DM12” is described as receiving E+12 vg/ml. However, Table 1 states that this group received 9.3E+11. Please clarify.

9. Figure 1 should show larger magnifications to appreciate individual neurons. Please describe if the mCherry was identified on cell bodies, dendrites and/or axons. Was there any evidence of transduction to non-neuronal cells?

10. Regarding the DAPI stain shown in figure 4: The images show in figure 4 reflect atrophy and other structural defects. However, these images should be presented side by side with images obtained from animals in the other groups. It should be clear that the defects seen in figure 4 are not due to mechanical damage done by the injections.

11. In the functional studies, it would be useful to explain what mechanism could underly the broader and distorted fEPSPs observed in the animals in groups DM12 and DM11

12. The Discussion section mentions that most previous studies rarely examine the expression of DREADDs after 18 weeks. (“No reports are available on long-term DREADD expression specifically in CaMKIIα-positive excitatory neurons", page 16). However, studies using DREADDs in non-human primates (NHPs) routinely examine expression several months (or years) after injection (see Eldridge et al 2016, Gray et al, 2016; Raper et al 2019, Upright and Baxter 2019, Nagai et al 2016). It makes me wonder that the time is not the only factor that can lead to detrimental effects of DREADD expression. I suggest that the authors comment on this and cite the NHP studies.

13. In the part of the Discussion dealing with previous reports of AAV-mediated toxicity, I suggest adding the report by Samaranch et al (2014).

Other minor concerns:

1. Introduction: “...but can instead be selectively activated by exogenous actuators such as clozapine-N-oxide and low doses of clozapine (Armbruster et al., 2007; Gomez et al., 2017).”. Mention, also, that DREADDs can be activated by deschloroclozapine (Nagai et al 2020) and JHU compounds (Bonaventura et al 2019)

2. Also in introduction, the titers and volumes that are ’reported in the literature” (bottom of page 2 and top of page 3) correspond to studies in rodents. Usually in non-human primate and other larger animals larger volumes are injected. Please clarify in text that these values are for small animals. Alternatively, add references and values for studies in larger animals.

3. Page 8: “except for the animal in which units were recorded”. Was only one animal used for single unit recordings? Please correct.

4. Add scale bars go panels E-L in figure 1

5. Indicate in figure 5 what are the ’multiple population spikes observed following hM4D(Gi) activation.

Reviewer 2: This is an interesting and timely paper. I think the experimental design is appropriate for the question. It is clear that the AAV titer significantly contributes to the effect observed. Moreover, The inclusion of a high titer AAV expressing only mCherry is critical. I had previously considered high expression of mCherry as a risk factor for cell death, and this is clearly not the case in this study, so that is important to show.

The methodology used to assess the impact of DREADD expression on hippocampus volume are appropriate, and it is reassuring to see consistent findings from both fMRI and post-mortem DAPI analyses. As the authors note in the discussion, the neuronal recording data is a little difficult to interpret. I am not sure what to make of this either, and but I think the authors did a good job of addressing this in the discussion.

Overall I think the question is important, the methodology is appropriate, and the analysis clearly shows the effects. I have two major comments/concerns:

1) Is this unique to the hippocampus? The hippocampus is known to be a brain region of neurogenesis, and is this one reason that these effects were observed? Do the authors think that comparable findings would be found in a cortical region? Is there any other evidence of this? Either additional experiments should be conducted, or the manuscript conclusions should be adjusted to specify this finding to the hippocampus.

2) The neural recording data is inconsistent between the different titers, but would behavioral manipulations also be inconsistent? Is it possible to run an additional experiment comparing high and low titer to a hippocampal dependent behavioral test?
